# Insect-borne non-enveloped bluetongue virus utilizes discrete small vesicles for non-lytic release and cell-to-cell transmission

**DOI:** 10.1371/journal.ppat.1013582

**Published:** 2025-10-09

**Authors:** Weining Wu, Ulrike Laugks, Kay Grünewald, Polly Roy

**Affiliations:** 1 Department of Infection Biology, Faculty of Infectious and Tropical Diseases, London School of Hygiene and Tropical Medicine, London, United Kingdom; 2 Centre for Structural System Biology, Hamburg, Germany; 3 Department of Chemistry, University of Hamburg, Hamburg, Germany; 4 Leibniz Institute of Virology, Hamburg, Germany; National Institute of Allergy and Infectious Diseases, UNITED STATES OF AMERICA

## Abstract

Bluetongue virus (BTV) is one of the most economically relevant orbiviruses and is the only example of a large complex, but non-enveloped arbovirus. In addition to cell lysis, BTV is known to employ a ‘budding’ process analogous to that used by enveloped viruses for cell exit, in which the viral glycosylated NS3 protein plays a key role. Recent reports have demonstrated that BTV can also induce non-lytic release via extracellular vesicles (EVs), however, details of the type and origin of the EV used and the role of NS3 in the process remain incompletely understood. In this study we undertook biochemical studies on the non-lytic release of BTV particles in different forms of EVs from several types of host cells and complemented this by comprehensive microscopic analyses using fluorescence microscopy, transmission electron microscopy and electron cryo-tomography. We discovered that BTV particles use both large EVs (LEVs) and smaller size EVs (SEVs) for non-lytic release and that, in each cell type studied, SEV fractions were particularly enriched with NS3. Non-enveloped BTV particles initially released in SEVs were highly infectious and promote efficient cell-to-cell transmission. This discovery highlights the complex mechanisms utilized by a non-enveloped arbovirus for egress and the significance of different EV types in this process.

## Author summary

BTV is an emerging arthropod-borne non-enveloped virus causing epidemics worldwide. The recent discovery that BTV and other non-enveloped viruses are secreted via EVs from infected cells, has opened a new window of opportunity to understand virus transmission and pathogenicity. Here, we used BTV, a complex multilayered virus, as a model to investigate the mechanism of virus-loaded EV formation and its effects on mammalian versus vector cells. Surprisingly, we discovered that in addition to large EVs (LEVs), BTV uses a small discrete size of EVs (SEVs) for efficient cell-to-cell transmission and that SEVs are enriched in the viral glycosylated NS3 protein when compared to particles associated with LEVs. Likewise, SEVs were also relatively more infectious, pointing to a role in transmission and pathogenicity. Our data identify two unique types of EVs associated with non-lytic release and the role of a non-structural viral glycoprotein in their formation.

## Introduction

Bluetongue virus (BTV) is a large (85 nm diameter) non-enveloped capsid virus, the prototype of Orbivirus, one of six genera of the family *Sedoreoviridae* (formerly *Reoviridae*), which includes vertebrate, arthropod, and plant pathogens [1]. Orbiviruses are typically arthropod-borne and comprise nineteen distinct species that are transmitted by mosquitoes, gnats and ticks. BTV, which is transmitted by the *Culicoides* vector (gnats), infects ruminants, often causing severe haemorrhagic disease in sheep and cattle. The virus particle has a complex icosahedral capsid structure of four consecutive protein layers. The innermost is the VP3 layer, which surrounds a polymerase complex of three proteins (VP1, VP4 and VP6) and a genome of ten double-stranded RNA segments, known as the subcore. The subcore is encapsidated by VP7 layer which forms a transcriptionally active core particle. The core is further encapsided by two additional consecutive protein layers, VP5 and the outermost layer VP2 both of which are responsible for virus entry into the host cells. In addition to seven structural proteins, the viral genome also encodes five non-structural proteins NS1, NS2 NS3/NS3A, NS4 and NS5 [2–5]. Due to the absence of a lipid envelope, the majority of mature BTV virus particles are typically released from infected mammalian cells by cell lysis in the late stage of infection [6]. However, emerging evidence has suggested that certain infected cells actively facilitate non-lytic release of newly synthesized virus particles prior to cell lysis. BTV has been observed to employ a similar ‘budding’ process mimicking that of enveloped viruses, in which the NS3 protein, the only glycosylated protein of BTV, plays a key role via a complex mechanism [7]. Moreover, a recent study showed that BTV particles are also released non-lytically in extracellular vesicles (EVs) from infected sheep cells by hijacking the host cellular secretion machinery, and that the EV associated virus particles are more infectious than free virus particles [8].

EVs are produced and released from almost all cell types for a wide variety of biological functions, most importantly as carriers of various cellular cargoes, such as proteins, nucleic acids and lipids. Primarily, two classes of EVs have been well-defined according to their sizes and origins, although a variety of other EV subtypes have also been discovered. The large extracellular vesicles (LEVs) are often known as microvesicles, approximately 0.2-2 µm in diameter, and are plasma membrane derived. The involvement of microvesicles in the non-lytic release of several non-enveloped viruses, such as rotavirus, coxsackievirus B and mammalian orthoreovirus, have previously been reported [9–11].

In contrast, non-enveloped small single-stranded RNA viruses, such as enterovirus 71 and norovirus, were also reported to induce non-lytic release via small extracellular vesicles (SEVs), which are derived from multi-vesicular bodies (MVBs), ranging in diameter from approximately 30–200 nm. They are commonly known as exosomes, characterized by carrying specific marker tetraspanin proteins like CD63, CD81 and CD9, and are released from the cells by an exocytic pathway after fusion of the MVB-bounding membrane with the plasma membrane [9,12]. Although it has been reported in principle that BTV can be released in EVs from infected sheep cells, many details remain unclear, particularly the type and origin of EVs utilized by BTV and the role of the viral glycosylated protein NS3 in this process. In this study, we undertook a comprehensive analysis combining biochemical studies of the process of non-lytic release of BTV from several types of host cells with various microscopic approaches to provide direct evidence of the nature and origin of the EVs involved. We discovered that the large multilayered complex BTV particles not only use LEVs but also SEVs for non-lytic release. In all three different types of cells tested, baby hamster kidney BSR cells (BHK-21 derived), natural host sheep kidney proximal tubules (PT) cells, and insect vector *Culicoides sonorensis*-derived (KC) cells, BTV proteins were found in both LEVs and SEVs and in all cell types, the NS3 protein was particularly enriched in SEVs, distinct from what had been reported for other viruses [9,13]. Both LEV- and SEV-associated viruses induced a more efficient infection than free virus particles, however, SEV-associated viruses were more efficient in infection than LEV-associated viruses. Transmission electron microscopy (TEM) visualisation of LEVs and SEVs being released from BTV infected BSR and PT cells also provided strong evidence on their nature, though gradual cell dependent differences were also observed.

This is the first report demonstrating that non-enveloped BTV particles are released in highly infectious SEVs, which in turn, enhance cell-to-cell non-lytic transmission of BTV. Our findings also highlight the complexity of the underlying mechanisms utilized by a non-enveloped arbovirus for egress and the significance of two types of EVs in this process.

## Results

### Cytoplasmic BTV particles associated with intracellular vesicles

**Fig 1:**
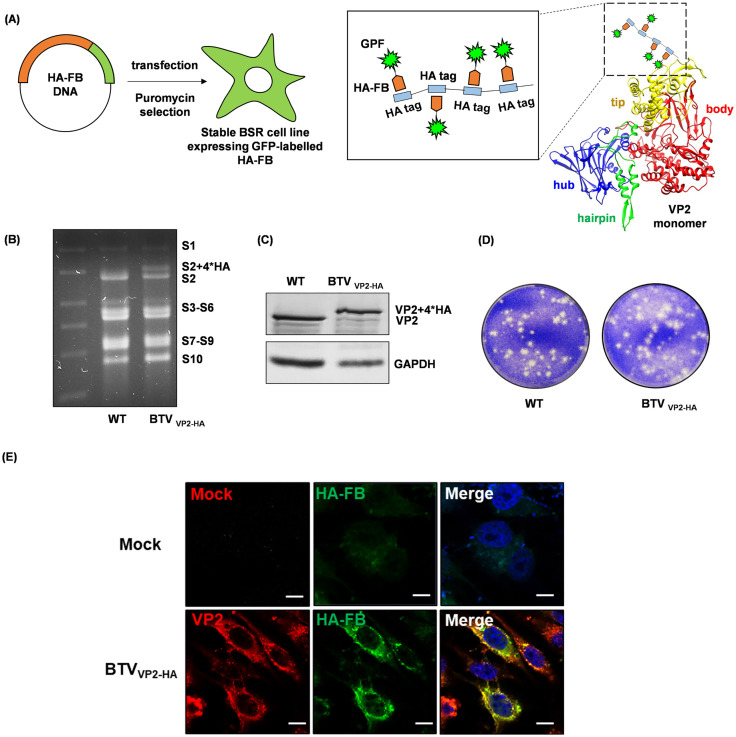
Generation of a stable BSR cell line expressing anti-HA-Franken bodies (HA-FB) and HA-tagged BTV virus. **(A)** Left: Cartoon showing that BSR cells were transfected with a plasmid expressing GFP-labelled HA-FB, and stably-transfected cells were selected using puromycin. Right: HA-tagged BTV virus was generated using reverse genetics by inserting four copies of the HA tag into the amino acid sequence between positions 218 and 219 of the VP2 tip domain, which allows binding of the HA-FB. **(B)** Comparison of 10 genomic RNA segments (S1-S10) between recombinant BTV_VP2-HA_ and wild-type virus by agarose gel electrophoresis. **(C)** Protein expression of VP2 in infected BSR cells at 24h pi analysed by western blot of recombinant BTV_VP2-HA_ vs. wild-type virus. **(D)** Comparison of plaque size between recombinant BTV_VP2-HA_ and wild-type virus. **(E)** Cytoplasmic VP2 labelled by anti-VP2 antibody (red, bottom row) can be specifically recognized by GFP-labelled HA-FB (green, bottom row) in BTV_VP2-HA_ infected BSR cells expressing HA-FB at 18h pi compared to the mock infected cells (top row). Colocalization appears yellow on merged images and nuclei stained with Hoechst are shown in blue. Scale bar = 10µm.

**Fig 2:**
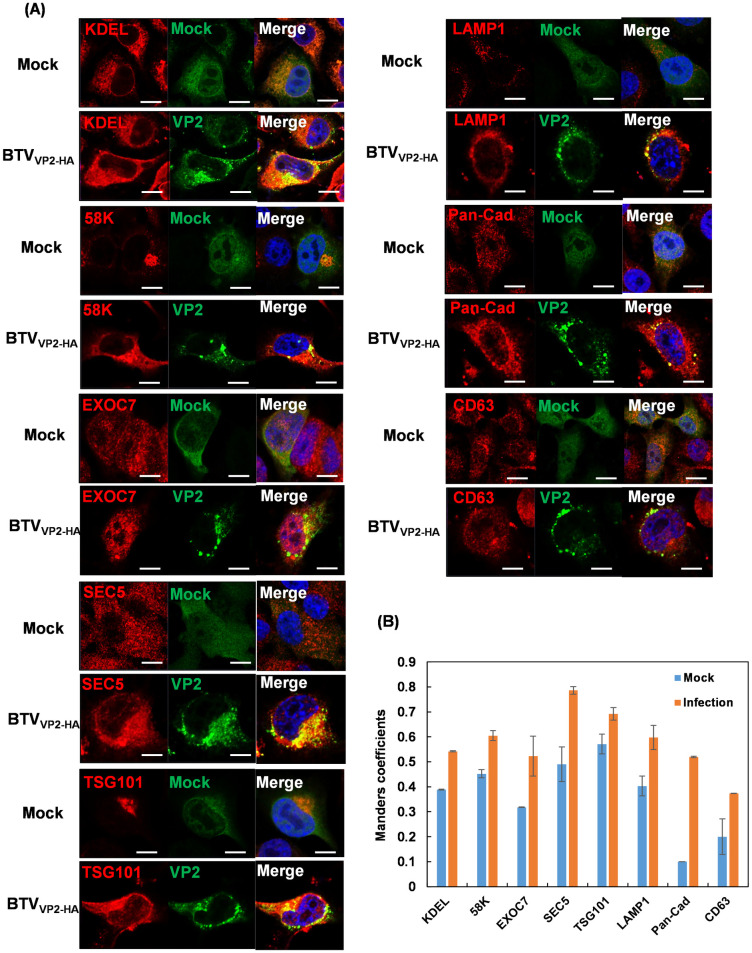
Colocalization of BTV outermost VP2 protein with selected markers of the exocytic secretion pathway by confocal imaging. Protein markers of the exocytic secretion pathway were labelled by specific antibodies shown in red. Cytoplasmic VP2 was visualized by GFP-labelled HA-FB in BTV_VP2-HA_ infected BSR cells expressing HA-FB at 18h pi, shown in green. Colocalization of BTV and cellular markers appear yellow on merged images. Nuclei were stained with Hoechst and shown in blue in the merged images. Scale bar = 10µm. **(B)** Bar chart representing results of colocalization quantification analysed using the JACoP plugin of ImageJ using Manders’ coefficients. A Manders’ overlap coefficients threshold above 0.5 was used to indicate a significant degree of colocalization.

BTV particles were previously observed within intracellular vesicles in infected host cells at late stages of infection [14,15]. Since most intracellular vesicles are produced in the endoplasmic reticulum (ER) and Golgi apparatus during the process of exocytosis, we wanted to perform colocalization analysis of the BTV outermost VP2 protein, a key player in the process of BTV assembly and egress, with selected markers of the exocytic secretion pathway by confocal imaging. Therefore, to enhance the visualisation of VP2 colocalization with cellular markers, we first generated a stable BSR cell line expressing green fluorescent protein (GFP)-labelled HA frankenbody (HA-FB) which has recently been developed by Zhao *et al* [16] to enable the precise binding of HA-tagged proteins (Fig 1A). In parallel, we also generated a HA-tagged BTV virus (BTV_**VP2-HA**_) using reverse genetics. We modified the VP2 gene by inserting four tandem copies of the HA tag into the amino acid sequence between positions 218 and 219 of the VP2 tip domain without compromising genome organization (Fig 1B), protein expression (Fig 1C) and virus replication (Fig 1D). The BSR cells expressing HA-FB were then infected with BTV_**VP2-HA**_ at an MOI of 1 for 18h. Cytoplasmic VP2 protein could specifically be recognized by GFP-labelled HA-FB (Fig 1E). As seen in Fig 2A, VP2 bound HA-FB displayed distinct subcellular distribution in contrast to unbound HA-FB in mock-infected cells. VP2 was observed to colocalize with a series of cellular markers of the exocytic secretion pathway including KDEL (ER marker), 58K (Golgi marker), and the EXOC7 and SEC5 components of the exocyst complex directing post-Golgi vesicle trafficking to the plasma membrane. Colocalizations between VP2 and TSG101, the marker for multivesicular bodies (MVBs), LAMP1 (lysosome marker) and pan-cadherin (plasma membrane marker), were clearly visible while colocalization of VP2 and the late endosome marker CD63 was barely visible, which is consistent with our previous observation by Du et al [17] (Fig 2A). In all cases except for CD63 the Manders’ Overlap Coefficient was above 0.5 (Fig 2B). The data indicate that the route of mature BTV particle transportation towards the plasma membrane is closely associated with the biogenesis of extracellular vesicles.

### Glycosylated NS3 facilitates intracellular vesicular trafficking of BTV

**Fig 3:**
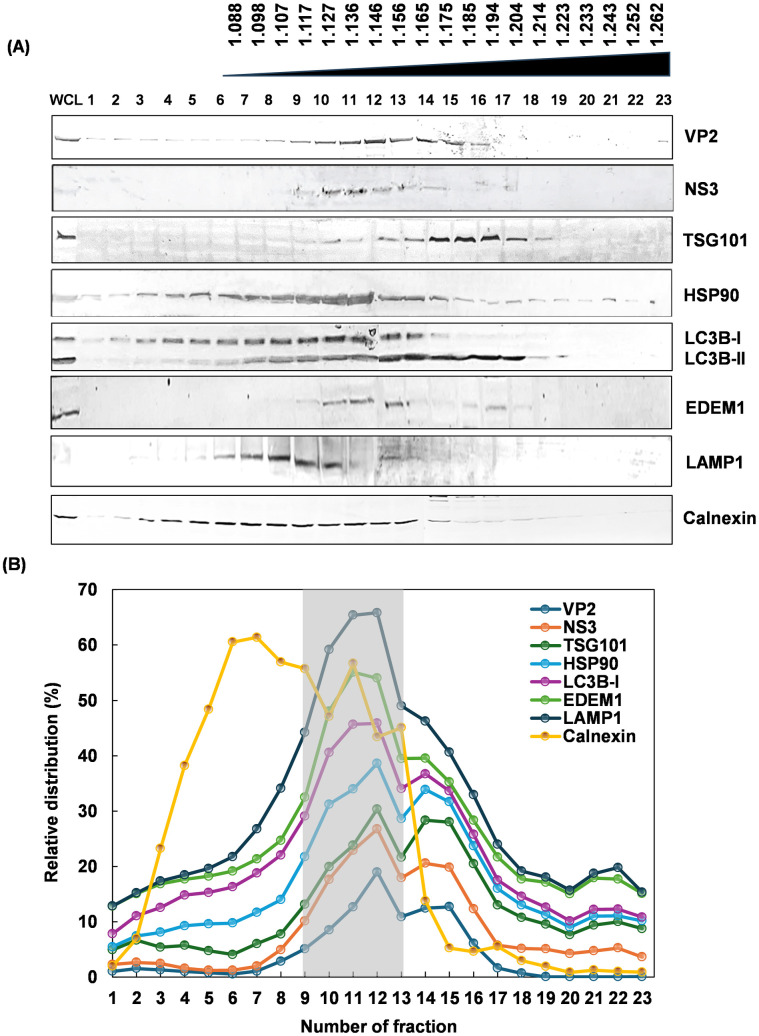
Co-migration of BTV proteins VP2 and NS3 with intracellular vesicle marker proteins by ultracentrifugation in an isopycnic iodixanol density gradient. **(A)** The supernatant of a whole cell lysate from infected BSR cells was loaded onto a linear 12-48% (w/v) Optiprep density gradient to allow separation of intracellular compartments and organelles. Both VP2 and NS3 were abundant in fractions 9 to 13 with a calculated range of density from 1.12 to 1.16 g/cm^3^. NS3 in particularly was not detected in other fractions. MVB-related proteins TSG101 and HSP90, autophagy related protein LC3B, ERAD-related protein EDEM1 and lysosomal membrane glycoprotein LAMP1 were also detected in the same fractions, except for Calnexin, an ER resident protein that is absent from intracellular vesicles. **(B)** Stacked line charts showing the quantification of relative abundance of each protein in different fractions by densitometry. Peak fractions 9-13 were highlighted in the light grey box.

BTV particle lacks any glycosylated protein, although the outer capsid protein VP2 has been shown to directly interact with the glycosylated NS3 [18]. To examine if NS3 is involved in BTV trafficking, BTV infected BSR cells were harvested at 24h pi, lysed by homogenization in a detergent-free buffer. Intracellular compartments and organelles were separated from whole cell lysate in an isopycnic iodixanol gradient by ultracentrifugation, and twenty-three fractions were subsequently collected from the top of the gradient. The presence of the membrane-associated NS3 protein in each fraction was detected by western blot using specific antibodies. Presence of NS3 was detected abundantly in fractions 9–13 (Fig 3A), the range of density was approximately 1.12 to 1.16 g/cm^3^, consistent with the buoyant density of EVs in iodixanol gradient [19]. Correspondingly, several intracellular vesicle protein markers of high interest, including the MVBs-related proteins TSG101 and HSP90, autophagy related protein LC3B, and ER-associated protein degradation (ERAD) related protein EDEM1, plus the lysosomal membrane glycoprotein LAMP1, were also examined. HSP90, LC3B-I and EDEM1 were found enriched in the same fractions alongside the viral proteins VP2 and NS3 in western blot, except for Calnexin, which is an ER resident protein generally absent from intracellular vesicles (Fig 3A) and confirmed by the measurement of relative distribution in the density gradient (Fig 3B), suggesting that NS3 is closely associated with the components of intracellular vesicles.

**Fig 4:**
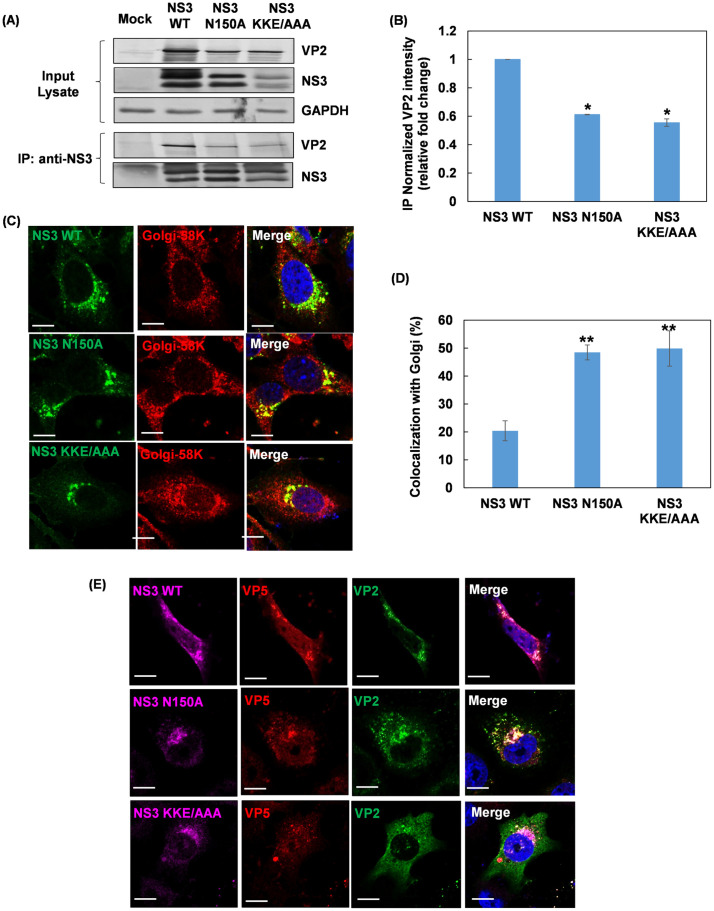
NS3 is vital for viral maturation and intracellular trafficking through its direct interaction with outer capsid proteins VP2 and VP5. **(A)** Co-IP analysis of the interactions between wild-type or mutant NS3 and VP2 from the cell lysates of BSR cells infected with wild-type or N150A and KKE_**196-198**_/AAA NS3 mutant viruses using an anti-NS3 antibody. GAPDH was used as control for equal protein loading. **(B)** Bar chart showing the normalized VP2 intensity measured by densitometry in Co-IP samples (right). Two-way ANOVA test *p < 0.05. **(C)** Confocal fluorescence microscopy of BSR cells infected with two NS3 mutant viruses showed Golgi retention of the N150A mutant NS3 and the KKE_196-198_/AAA mutant NS3. **(D)** Bar chart showing the percentage of colocalization between NS3 and Golgi (right). Two-way ANOVA test *p < 0.05. **(E)** Confocal microscopy showed that N150A mutant NS3 and the KKE_196-198_/AAA mutant NS3 no longer colocalized with VP2 and VP5 compared to the wild-type NS3. Both mutations in the NS3 resulted in a loss of VP2 and VP5 plasma membrane trafficking compared to wild-type NS3 (bottom row). Colocalization appears yellow on merged images. Scale bar = 10µm.

The co-migration of NS3 with these intracellular vesicle marker proteins in the density gradient, and the interactions previously found between NS3 and multivesicular body related proteins NEDD4 and TSG101 indicated a link between NS3, the only viral transmembrane glycoprotein, and intracellular vesicles [14]. Moreover, NS3 was observed to colocalize with the outer capsid VP2 and VP5 proteins, in close association with the viral inclusion bodies (VIBs, site of virus assembly) in the early hours (6 to 8h) of post-infection (S1 Fig), consistent with previous studies [7,18,20]. Therefore, the involvement of NS3 in virion maturation was further investigated using two NS3 mutant viruses generated by reverse genetics. The N150A mutation removed the only glycosylate site of the NS3, and the KKE_**196–198**_/AAA mutation at the C-terminus of the NS3 weakened the interaction between NS3 and VP2 [7], which was confirmed by a Co-immunoprecipitation (Co-IP) experiment (Fig 4A) and as expected, the amount of VP2 bound to mutant NS3 was significantly decreased (Fig 4B). Further, confocal fluorescence microscopy analysis showed a pattern of retention of both mutant NS3 within the Golgi apparatus (Fig 4C) with a significantly increased colocalization with Golgi (Fig 4D), and that the KKE_**196–198**_/AAA mutant NS3 no longer colocalized with VP2 and VP5, compared to the wild-type NS3. More importantly, plasma membrane trafficking was severely impaired for both mutant viruses (Fig 4E), emphasising that NS3 is vital for intracellular trafficking of BTV particles.

### BTV particles are released in NS3 enriched SEVs

**Fig 5:**
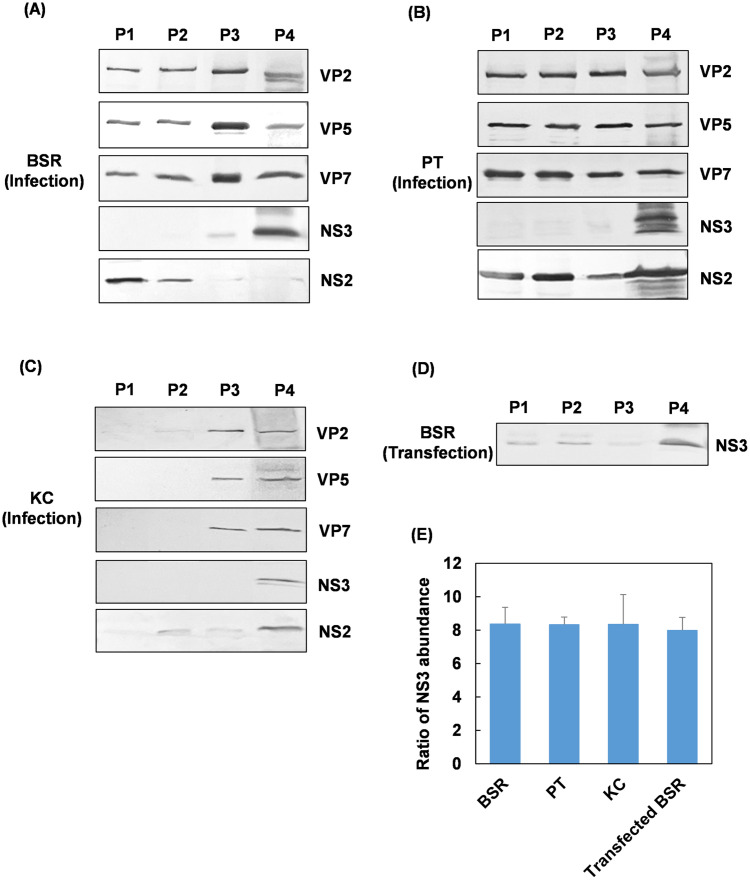
Isolation and characterization of EVs released from BTV infected mammalian. (A) BSR cells, (B) sheep PT cells and (C) insect KC cells, or (D) NS3-transfected BSR cells. Four pellets were isolated from the cell culture supernatant by differential centrifugation at 300xg (P1), 2000xg (P2) and 10,000xg (P3) followed by PEG-10,000 precipitation (P4), containing detached cells (P1), dead cells (P2), large extracellaule vesicles (LEVs) (P3) and small extracellular vesicles (SEVs) (P4), respectively, and resuspended in equal volume of PBS buffer. The abundance of viral capsid proteins VP2, VP5 and VP7, and nonstructural proteins NS3 and NS2 in P1-P4 from all three cell lines infected with BTV **(A-C)**, or the abundance of NS3 in P1-P4 from NS3-transfected BSR cells were analyzed by western blot. **(E)** Bar chart showing the ratio of NS3 abundance in SEVs compared to that in LEVs from (A-D) measured by densitometry.

**Fig 6:**
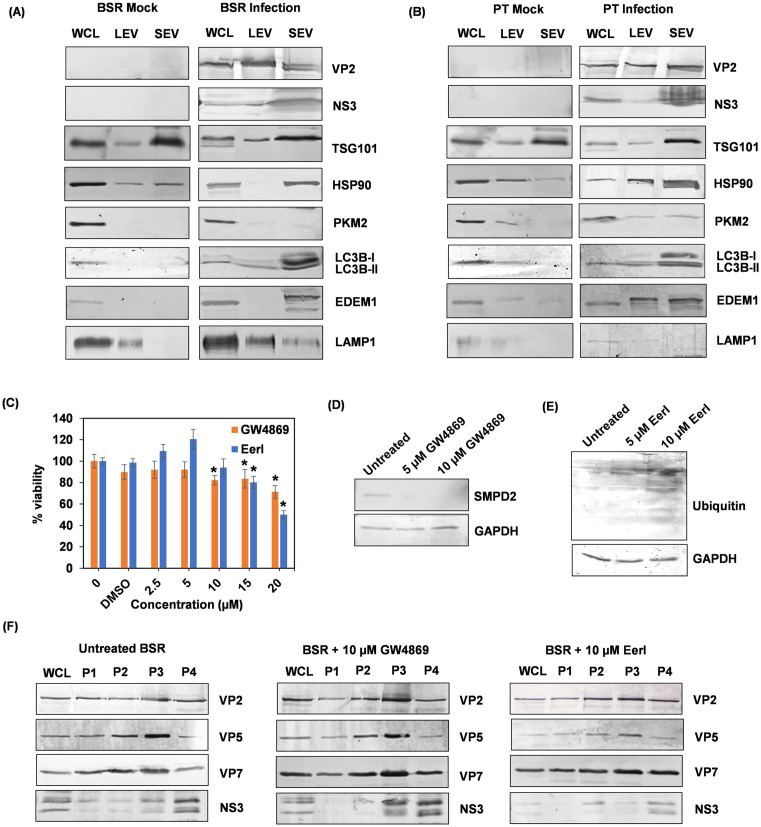
Comparison between LEVs and SEVs of their different origins and compositions, and the inhibitory effect of different inhibitors on the release of virus in EVs by western blot. The viral proteins VP2 and NS3, and a series of subcellular organelle protein markers were analyzed against the LEVs and SEVs isolated from mock infected or BTV infected BSR (A) and PT (B) cells. Whole cell lysate (WCL) was included as a positive control. **(C)** MTT cytotoxicity assay measures cytotoxic effect on BSR cells at the concentrations of GW4869 and EerI used. Two-way ANOVA test *p < 0.05. **(D)** The expression of sphingomyelin phosphodiesterase 2 (SMPD2), which is inhibited by GW4869 treatment, and (E) ubiquitinated proteins on EerI treatment were analyzed by western blot. GAPDH was used as control for equal protein loading. (F) compared to untreated BSR cells (left), the treatment of 10 µM of GW4869, a potent exosome inhibitor, failed to inhibit the release of SEVs (P4) from BTV infected BSR cells (middle). BTV infected BSR cells treated with EerI, which blocks the process of moving misfolded proteins from the ER to cytoplasm for ubiquitin-mediated protein degradation, resulted in decreased abundance of intracellular NS3 and NS3 in SEVs (P4) (right).

Although several non-enveloped viruses including BTV have been reported to be released from EVs, BTV is the only non-enveloped virus that is also known to mediate its non-lytic release through a ‘budding’ process [21]. To characterize the EVs released from BTV infected mammalian or insect cells, BSR, PT and KC cells were infected with BTV at an MOI of 1, 10 and 20 respectively, and incubated for 24h. Dead cells and EVs were then separated from the supernatants of infected cells by differential centrifugation methods and PEG-10,000 precipitation. Four different pellets were isolated from the cell culture supernatant, as follows: P1, the detached cells; P2, the dead cells; P3, the LEVs; and P4, the SEVs. The protein content of each pellet was analyzed by western blot after protein separation by SDS-PAGE. Viral capsid proteins VP2, VP5 and VP7 were present in all four pellets obtained from BSR and PT cells (Fig 5A & 5B), while for vector KC cells, viral proteins could only be detected in P3 and P4 (Fig 5C). Remarkably, the NS3 viral protein was present significantly more abundance in the P4 isolated from all three cell lines, and was approximately 8 times more abundant in SEVs than LEVs measured by densitometry (Fig 5E), while the control NS2 protein did not show a similar pattern, suggesting that NS3 is uniquely high in the SEVs (Fig 6A & 6B & 6C). Moreover, NS3 was also found similarly enriched in SEVs derived from BSR cells transfected with a plasmid expressing only the NS3 protein, in the absence of other viral proteins (Fig 5D and 5E). These data indicate that NS3 may be involved in the pathways of the SEVs biogenesis.

These differential characteristics of SEVs from LEVs, apart from their size, are suggestive of their different origins and compositions. To investigate further the differences between the SEVs and the LEVs, a series of subcellular organelle markers were analyzed against the LEVs and SEVs isolated from mock infected or BTV infected BSR and PT cells by western blot analysis using specific antibodies. In both cell types, compared to the LEVs, which was reported to originate from the autophagy-lysosomal pathway (ALP) [8], the SEVs were highly abundant with MVBs markers TSG101 and HSP90, and viral NS3 protein (Fig 6A and 6B), indicating their endosomal origin. HSP90 is a regulator of MVBs, stabilizing TSG101 to increase the number of endosomes and EVs [22]. However, due to the absence of late endosome/exosome marker PKM2, these endosome-derived SEVs are unlikely to be exosomes. To further validate this, BSR cells were treated with 10μM of GW4869, a potent exosome inhibitor at 1h post-infection, which showed no cytotoxic effect in an MTT assay (Fig 6C), was capable to inhibit neutral sphingomyelinase-2 (SMPD2), the enzyme that plays a crucial role in exosome formation [23] (Fig 6D). The failure of GW4869 to inhibit the release of SEVs in both BSR (Fig 6F) and PT cells [8] confirmed that these endosome-derived SEVs are not exosomes. Interestingly, the non-lipidated autophagy related protein LC3B-I, and ER-associated protein degradation (ERAD) related protein EDEM1, required for the formation of ER-derived EDEMosomes or double-membrane vesicles (DMVs), were also found in abundance in the SEVs from only BTV infected BSR and PT cells but not in those from mock infected cells (Fig 6A and 6B). EDEMosomes or DMVs are small vesicles similar to the autophagosomes with a size of approximately 100–300nm in diameter and they are produced independent of the autophagic pathway through the endoplasmic reticulum (ER)-associated degradation (ERAD) machinery [24,25]. Therefore, the presence of these markers in the SEVs suggests that they could originate from the fusion between MVBs and EDEMosomes/DMVs and are subsequently transported to lysosomes for the release of virus particles. The abundance of viral protein NS3 in SEVs suggests that this process likely to be regulated by the NS3 during BTV infection. BTV infected BSR cells at 1h post-infection, were treated with 10μM of Eeyarestatin I (EerI), a potent inhibitor of endoplasmic reticulum associated protein degradation (ERAD). The treatment showed no cytotoxic effect on BSR cells by MTT assay (Fig 6C), however, it enabled to block protein translocation from ER to cytoplasm for ubiquitin-mediated protein degradation, leading to the accumulation of ubiquitinated proteins (Fig 6E). Moreover, EerI treatment showed a significant reduction of the intracellular NS3 level as well as in the SEVs, although none of the other viral structural proteins were affected (Fig 6F).

**Fig 7:**
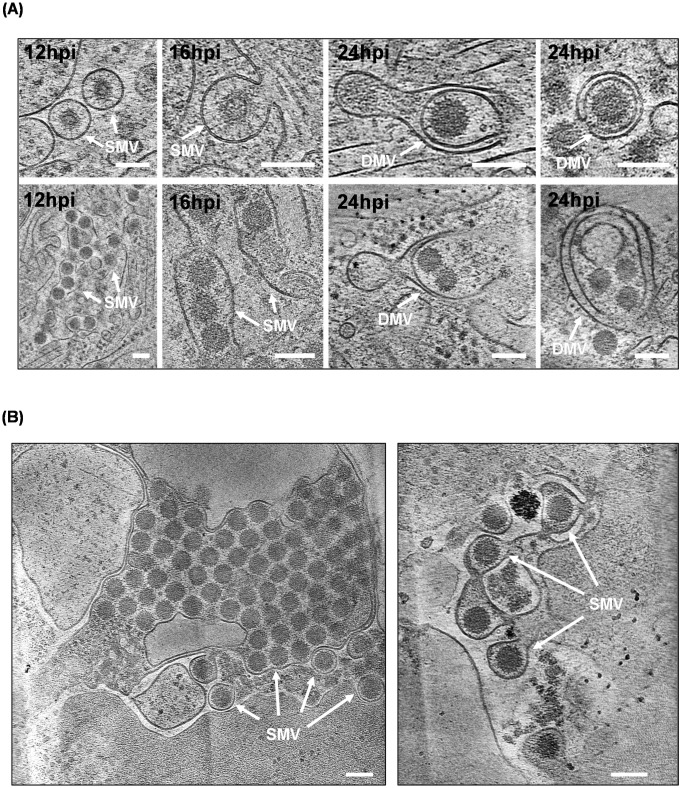
Cryo-ET visualization of BTV infected BSR cells. **(A)** At early time points (12h and 16h pi) lamellae of subcellular regions showed that many virus particles were observed within small single membrane vesicles (SMVs), typically containing only a single particle (top row, 1^st^ and 2^nd^ columns), while large SMVs contain multiple virus particles (bottom row, 1^st^ and 2^nd^ columns). In contrast, at 24h pi many virus particles were observed within double membrane vesicles (DMVs), containing single (top row, 3^rd^ and 4^th^ columns) or multiple virus particles (bottom row, 3^rd^ and 4^th^ columns). **(B)** Panels showing unmilled areas of the cell periphery with singular or multiple virus particles in SMVs being released from the cell. Scale bar = 100nm.

**Fig 8:**
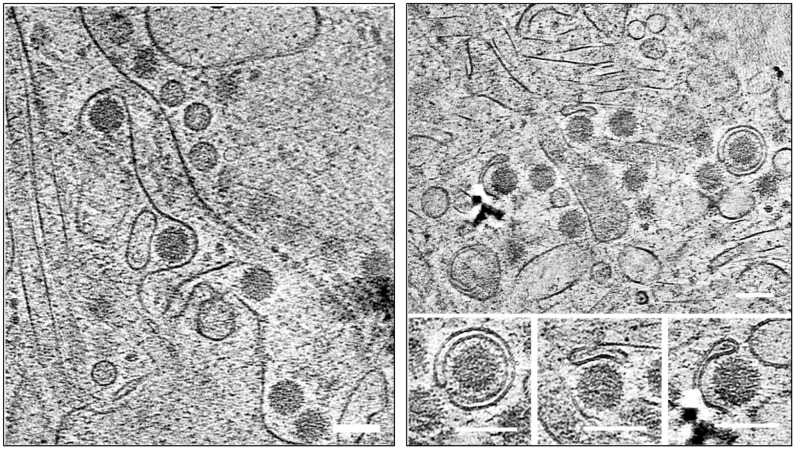
Cryo-ET FIB-milled lamellas show the presence of viral particles in EDEMosomes/DMVs in BTV infected BSR cells at 24h pi. The left panel shows viral particles making contact with the ER. The right panel shows snapshots of the different stages of viral particles being engulfed in EDEMosomes/DMVs. Scale bar = 100nm.

To gain more direct evidence and a better understanding of the interactions between intracellular vesicles and virus particles during BTV infection, BSR cells were seeded onto electron microscopy grids followed by infection with BTV at an MOI of 3 for 12h and 16h, or an MOI of 1 for 24h. Cells were plunge frozen in liquid ethane/propane mixture, and the thin lamellas were prepared by Focused Ion Beam (FIB) milling for high-resolution tomographic imaging using a cryo-transmission electron microscope (cryo-TEM). At the early time points, 12h and 16h pi, many virus particles were observed within intracellular single-membraned vesicles (SMVs), some were large vesicles containing multiple virus particles, while others were small vesicles, each containing only one single particle (Fig 7A). In contrast, at 24h pi, single and multiple virus particles could be visualised within double-membraned vesicles (DMVs), which resemble small autophagosomes (Fig 7A). Moreover, cryo-ET applied on the unmilled thinner cell periphery of BSR cells infected with BTV at 24h pi exhibited not only a large number of virus particles within large SMVs, but also individual virus particles within small SMVs that appear to be budding off from the plasma membrane (Fig 7B), suggesting that virus particles are trafficked within intracellular vesicles to the cell membrane prior to release. In addition, viral particles engulfed in ER-derived EDEMosomes/DMVs were also observed in BTV infected BSR cells at 24h pi by cryo-ET (Fig 8). A tomographic slice reveals viral particles closely interacting with ER membranes through fine, connector-like structures bridging the gap between virions and membrane surfaces. Both small and extensive ER membrane patches are seen surrounding the particles, with stretches of ER wrapping around individual virions. In several instances, smaller patches of ER membrane tightly enclose single particles, reshaping into DMVs, as shown in various stages in the right panel of Fig 8. The small diameter of these DMVs of less than 200nm suggests that this process is distinct from classical autophagy and represents the formation of EDEMosomes originating from ER-derived membranes.

### SEVs-enclosed BTV particles induce a more efficient infection

**Fig 9:**
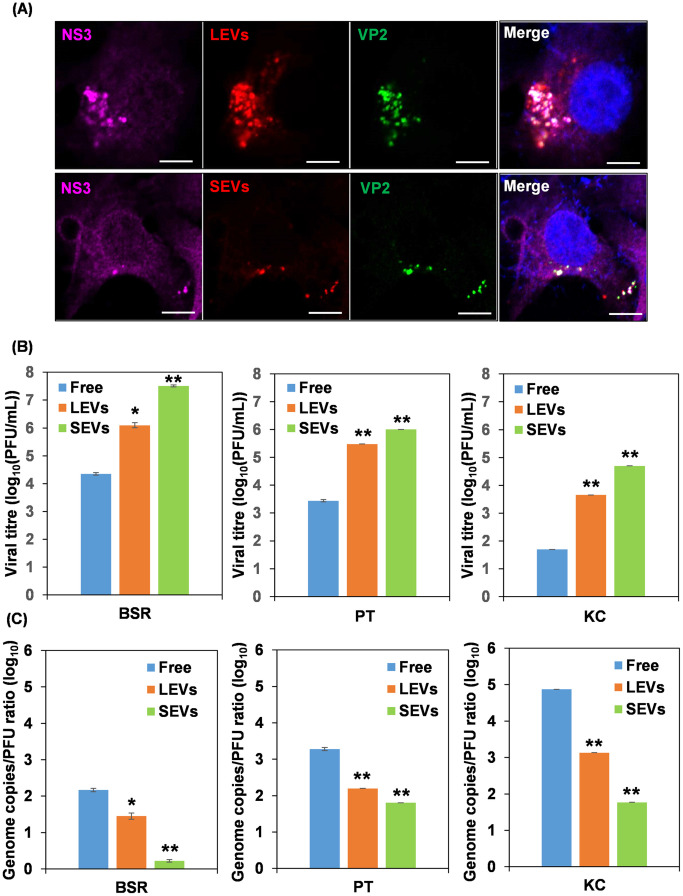
Viral particles in SEVs induce efficient infection. **(A)** Confocal imaging showed that both LEVs and SEVs, labelled with PKH26 red fluorescent dye, were internalized by BSR cells, and were colocalized with viral proteins VP2 and NS3 (shown in green and purple, respectively) in EV uptake experiments. Colocalization appears yellow on merged images and nuclei stained with Hoechst are shown in blue. Scale bar = 5µm. **(B)** Titre comparison of equal volume of LEVs and SEVs diluted in PBS and free supernatant virus collected from BTV infected BSR, PT and KC cells at 24h pi. **(C)** Comparison of the specific infectivity of LEVs, SEVs and free supernatant virus calculated as genome copies-to-PFU ratio in BSR, PT and KC cells. Two-way ANOVA test *p < 0.05, **p < 0.01.

To investigate whether BTV particles within the SEVs are relevant to virus transmission efficiency and infectivity, LEVs and SEVs isolated from BTV infected BSR cells were labelled with the PKH26 red-fluorescent lipophilic dye, which stains membranes by intercalating its aliphatic portion into the exposed lipid bilayer [26]. EV uptake experiments were then undertaken by incubating the fluorescent labelled EVs with BSR cells for 1h before cells were fixed and labelled with antibodies against viral proteins. Confocal imaging demonstrated that both LEVs and SEVs were actively internalized by BSR cells, and were colocalized with viral proteins VP2 and NS3 (Fig 9A), suggesting that both LEVs and SEVs can mediate virus transmission from cell to cell. Moreover, the virus titres of LEVs, SEVs and supernatant collected from BTV infected BSR, PT and KC cells were determined by plaque assay, and the number of particles based upon viral genome copy number was determined by reverse transcription-quantitative PCR (qRT-PCR). The titre of SEVs was significantly higher compared with that of LEVs and free viruses in supernatant of all three type of cells (Fig 9B). When the specific infectivity of SEVs, calculated as the genome copies-to-PFU ratio, was compared with that of LEVs and free viruses, SEVs displayed a significantly lower genome copies-to-PFU ratio in contrast to LEVs and free viruses in all three types of cells. The fold decrease of SEVs versus LEVs was 0.06, 0.4 and 0.04 in BSR, PT and KC cells respectively, while the fold decrease of SEVs versus free viruses was 0.01, 0.03 and 0.001 in BSR, PT and KC cells respectively (Fig 9C). These data suggest that SEVs-enclosed viruses are more infectious, and BTV may take advantage of these NS3 enriched SEVs to deliver a more efficient infection during cell-to-cell transmission.

### EM analysis shows that SEVs are specific for efficient virus transmission

**Fig 10:**
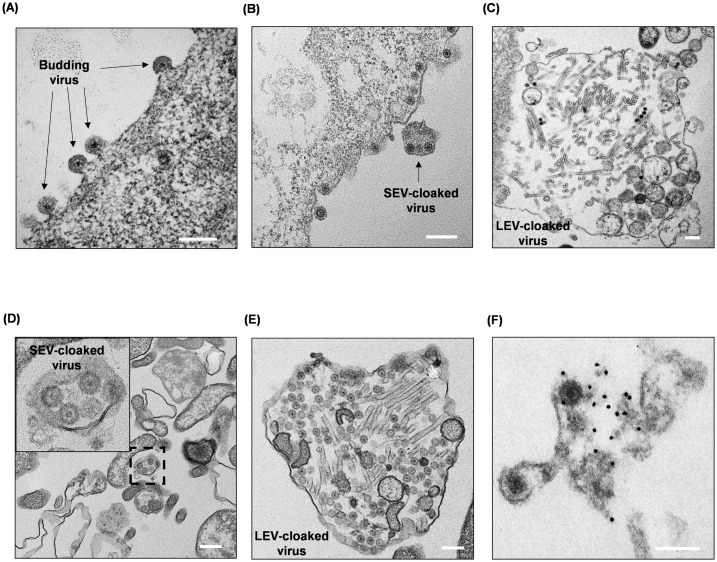
Transmission electron microscopy (TEM) on ultrathin sections of BTV infected BSR and PT cells at 24h pi. (A) most virus particles were released by non-lytic viral budding in BTV infected BSR cells, although virus particles were also observed to be released in SEVs (B) or LEVs (C) from BTV infected BSR cells but were not predominant. **(D)** SEVs released from BTV infected PT cells contain only a few virus particles. **(E)** LEVs released from BTV infected PT cells contain not only multiple virus particles but also other intracellular materials. (F) immunogold labelling with NS3 antibody showed enrichment of NS3 in SEVs released from infected PT cells. Scale bar = 200nm.

To support the above biological data by electron microscopy visualization BSR and PT cells were infected with BTV at an MOI of 1 and 10 for 24h before entering a predominant cell lysis stage. Ultrathin sections of infected cells were prepared for TEM. In BTV infected BSR cells, the majority of virus particles were observed to be released by non-lytic unpolarized viral budding (Fig 10A). Although virus particles released in SEVs and LEVs could also be visualized, they were not as predominant as the budding viruses in BSR cells (Fig 10B and 10C). In contrast, virus particles released in EVs were predominant in BTV infected PT cells, and no obvious budding events were observed (Fig 10D & 10E), consistent with a previous report [14]. Instead, the SEVs observed approximately 200nm in diameter, contain only one or a few virus particles (Fig 10D), and the presence of NS3 in the SEVs was further detected by the NS3 antibody immunogold labelling, however the altered morphology suggested that SEVs were structurally fragile and susceptible to damage during the process of immunogold labelling (Fig 10F). In contrast to SEVs, LEVs measured nearly 2µm in diameter, encompass not only multiple virus particles, but also other intracellular materials, such as BTV tubules, transport vesicles, and cytoskeletons, suggesting that they are likely to involve in transferring intracellular cargos (Fig 10E). The distinct difference of the contents and compositions between SEVs and LEVs suggested that the SEVs are possibly produced more specifically in response to BTV infection, therefore are more efficient for virus cell-to-cell transmission.

## Discussion

Although cell lysis is the primary mode of host cell exit for non-enveloped viruses, many non-enveloped viruses have also been observed to actively facilitate non-lytic release via extracellular vesicles, such as picornaviruses including poliovirus and hepatitis A virus (HAV) [27,28], rotaviruses belonging to the family *Sedoreoviridae* [9], and orthoreovirus belonging to the family *Spinareoviridae* [11]. BTV is also a member of the family *Sedoreoviridae*, however it belongs to the genus *Orbivirus* and like other orbiviruses, is an arbovirus which is transmitted from insect vector to animal host. Although BTV is non-enveloped, it encodes a unique membrane glycoprotein NS3. BTV was previously reported to induce non-lytic exit through a budding process similar to enveloped viruses with the participation of the NS3 protein, in a manner different from rotavirus [29]. It has also been recently reported that BTV is released in EVs from infected sheep cells [8], suggesting that BTV takes advantage of more diverse non-lytic egress pathways than was believed. However, the underlying mechanisms and importance for viral pathogenesis in relation to egress are not well understood.

Using high-resolution cryo-ET and confocal microscopy, we showed in this study that within BTV infected BSR cells multiple virus particles are transported via intracellular vesicles following their release from the viral inclusion bodies (VIBs), the site of viral assembly, to the cell periphery. At 12-16h pi, both large SMVs containing multiple virus particles or small SMVs containing only a single virion were present. In contrast, DMVs containing single or a few virions only occurred at the late stage of infection (24 h pi). The DMVs, or EDEMosomes, similar to autophagosomes albeit smaller, are ER-derived double-membrane vesicles, and are often produced during infection of single-stranded positive sense RNA (+ssRNA) viruses, such as enveloped coronavirus and hepatitis C virus (HCV) [24,30]. These viruses hijack membranes of the secretory and autophagy pathways and trigger a rearrangement of ER membrane to form the SMVs, and later DMVs/EDEMosomes, known as viral replication organelles (ROs) containing the viral replication intermediates, for active viral genome replication [31]. Viral non-structural proteins have been identified to play a critical role in DMVs biogenesis, such as coronavirus nsp3 and nsp4 [32], poliovirus 2BC and 3A [33]. In the case of norovirus NS4 and HCV NS5A, a single non-structural protein is sufficient for the formation of DMVs [34,35]. BTV does not form membranous ROs for viral genome replication, instead the association of VP2 and particularly NS3 with the MVBs and DMVs/EDEMosomes observed in the density gradient suggested that BTV could also hijack the host autophagic and ERAD machineries. It is possible that NS3 triggers ER remodelling/vesicle formation to associate with the VIBs, thereby facilitating the export of viral core particles, which may have already been encapsidated by the outer capsid protein(s) at the periphery of VIBs, from the VIBs to the site of release via intracellular DMVs.

**Fig 11:**
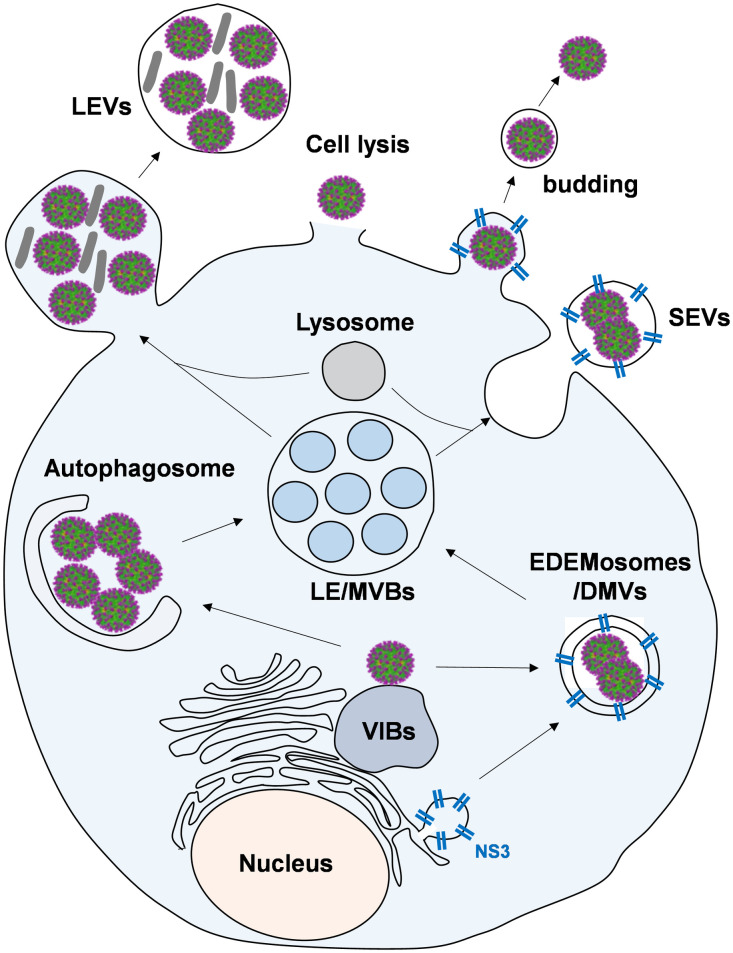
Cartoon showing the hypothesis of the origin of SEVs. Viral NS3 protein triggers ER remodelling for the formation of NS3-enriched ER-derived double-membrane vesicles (DMVs)/EDEMosomes. SEVs could originate from the fusion between MVBs and these DMVs/EDEMosomes to promote more efficient cell-to-cell virus transmission in addition to the other pathways of virus egress previously described.

Analysis of the protein compositions of extracellular LEVs and SEVs highlighted the similar composition of SEVs with MVBs and DMVs/EDEMosomes, suggesting that the SEVs could originate from the fusion between MVBs and DMVs/EDEMosomes, as described in the cartoon (Fig 11). Moreover, we showed that the SEVs associated viruses are more infectious with lower genome copies-to-PFU ratios than those in LEVs and those of free viruses. A significantly higher amount of NS3 was also found in the SEVs. NS3 is known to directly interact with TSG101 and NEDD4 which are required for the formation and sorting process of MVBs [29]. NEDD4 is also involved in autophagic processes through the interaction with LC3, therefore it may connect endosomal vesicle trafficking and autophagic processes, thereby coordinating the endosome-dependent and the autophagosome-dependent lysosomal degradation process [36]. The involvement of NS3 in SEV formation suggests they are generated more readily during BTV infection, when compared to the LEVs, used for general cargo transport between cells.

Although BTV can be released in EVs from both mammalian and insect cells, this non-lytic EV mode is more predominant in the natural host sheep cells rather than the lab-adapted BSR cell line and *Culicoides* vector cells. This could potentially benefit BTV infection from several aspects such as delivering multiple viral genomes, so called ‘collective infectious units’ to the recipient cells simultaneously, a more rapid receptor-independent cell-to-cell transmission or evasion from host immune response. Additionally, from an evolutionary point of view, non-enveloped viruses are more resistant to extreme pH, heat, desiccation and various disinfectants due to the lack of a lipid envelope in their structures. If it is assumed that their ancestor viruses were enveloped, non-enveloped viruses have developed complementary non-lytic exit strategies regulated by specific viral proteins after losing their envelopes. However, further investigations remain necessary such as the cell type-dependent differences and the possible role of arthropod vectors in the adaptation of non-enveloped arboviruses during their evolution [37].

## Materials and methods

### Cell lines and virus

Baby hamster kidney BHK21-derived BSR cells, and sheep kidney proximal tubules (PT) cells were maintained in Dulbecco’s Modified Eagle’s Medium (DMEM)-high glucose (Sigma Aldrich), supplemented with 5% foetal bovine serum (FBS, Gibco), 1X non-essential amino acids (NEAA, Gibco, PT cells only) and antibiotic-antimycotic (100 units/mL of penicillin, 100 µg/mL of streptomycin and 0.25 µg/mL of amphotericin B, Gibco) at 35°C in a humidified 5% CO_2_ incubator. *Culicoides sonorensis*-derived KC cells were maintained in Schneider’s Insect Medium (Sigma), supplemented with 10% FBS and antibiotic-antimycotic at 28°C.

BTV serotype 1 (BTV-1) virus stock was obtained by infecting BSR cells at a low multiplicity of infection (MOI), and viral supernatant was harvested at approximately 48h to 72h post-infection (pi) when cytopathic effect (CPE) became evident. Virus stock was stored at 4°C, and the titre was determined by plaque assay as previously described [38], expressed as plaque forming unit (PFU)/mL.

### Cryogenic electron tomography (cryo-ET)

Gold grids with SiO_2_ film (Quantifoil, Au 200 mesh, R 1/4) were glow-discharged for 45 sec in a GloCube (Quorum) and coated with 20µg/ml fibronectin solution for 30min. Trypsinized BSR cells were applied to grids in 25µl culture medium at a density of 3x10^3^ cells/grid. Grids were incubated in ibidi dishes for 12h. BTV was added to the cells and incubated for 12 and 16h at an MOI of 3, or 24h at an MOI of 1. Samples were plunge-frozen with Leica GP2 plunger in liquid ethane propane mixture (37%/63%).

Vitrified BSR cells infected with BTV were kept under cryo conditions at all times. For sample transfer to the cryo focused-ion beam scanning microscope, grids were clipped in autogrids modified for FIB-milling (custom made) and transferred to an Aquilos FIB/SEM (Thermo Fisher Scientific). Lamella of a thickness between 120nm and 200nm were prepared using automated milling software AutoTEM (Thermo Fisher Scientific) and manual lamella polishing.

After FIB-milling, samples were transferred to a Titan Krios transmission electron microscope working under cryo conditions (Thermo Fisher Scientific). The 300kv TEM was equipped with a field emission gun (XFEG) and a Gatan Bioquantum energy filter with a slit width of 20eV together with a Gatan K3 direct electron detector. Lamella of BTV infected BSR cells were screened for viral particles at an intermediate magnification of 3600x. Tilt series were acquired using SerialEM software with total dose of 100–120 e^-^/Å^2^ and total tilt range of 120° with the starting angle of relative 0° in respect to the lamella milling angle. Tilt series were acquired in dose symmetric tilt scheme with a group size of 2 and tilt increment of 3°. Data was acquired at a defocus of 5µm and at nominal magnification of 26000x and pixel size of 3.336Å. Tilt series were aligned and reconstructed by weighted back projection using IMOD Etomo or AreTomo at a binned pixel size of 13.344Å.

### Generation of HA-tagged virus and stable cell line expressing HA-FB

HA-tagged BTV virus (BTV_VP2-HA_) was generated by inserting four consecutive human influenza hemagglutinin (HA) tags (YPYDVPDYA) between aa218 and aa219 of the VP2 open reading frame (ORF) by site-directed mutagenesis (Forward primer: 5’-GATCAGACATTAATTAATTTTTACCCATACGATGTTCCAGATTACGCTGGCTACCCATACGATGTTCCAGATTACGCTGGATACCCATACGATGTTCCAGATTACGCTGGCTACCCATACGATGTTCCAGATTACGCTGGGAGAGGTCAGAAGGTGGC-3’; Reverse primer: 5’-GCCACCTTCTGACCTCTCCCAGCGTAATCTGGAACATCGTATGGGTAGCCAGCGTAATCTGGAACATCGTATGGGTATCCAGCGTAATCTGGAACATCGTATGGGTAGCCAGCGTAATCTGGAACATCGTATGGGTAAAAATTAATTAATGTCTGATC-3’). The mutant virus was recovered by reverse genetics as previously described [39]. To generate the stable cell line expressing HA-FB, BSR cells were transfected with a plasmid pCAG-15F11-HA-mEGFP (adapted from pCMV-15F11-HA-mEGFP, Addgene #129590) expressing HA-FB. Stably-transfected cells were selected and maintained in growth medium containing 7.5µg/ml of puromycin (Gibco) 48h post-transfection.

### Immunofluorescence

A monolayer of BSR cells grown overnight on coverslips were infected with BTV-1 at an MOI of 1 for 24h, or an MOI of 10 for 6-8h. Infected cells were fixed in 4% paraformaldehyde, and then permeabilized with 0.1% Triton X-100 in phosphate-buffered saline (PBS). Cells were labelled with anti-NS3, anti-VP5 and anti-VP2 (in-house antibodies), or anti-KDEL (Abcam, ab12223), anti-58K (Abcam, ab27043), anti-EXOC7 (Abcam, ab95981), anti-SEC5 (Proteintech, 12751–1-AP), anti-TSG101 (Abcam, ab30871), anti-CD63 (Abcam, ab118307), anti-LAMP1 (Abcam, ab24170), and anti-pan-cadherin (Abcam, ab22744) primary antibodies, followed by the appropriate Alexa fluor secondary antibodies (Thermo Fisher Scientific). Nuclei were stained with Hoechst. Images were captured using the LSM 880 inverted confocal microscope (Carl Zeiss Ltd.).

### Iodixanol gradient ultracentrifugation

BSR cells were infected with BTV-1 at an MOI of 1 for 24h. Cells were carefully washed with ice-cold PBS (pH 7.4), harvested with a cell scraper, and lysed in homogenization buffer (10mM Tris-HCl pH 7.4, 1mM EDTA, 250mM sucrose, protease inhibitor cocktail) in an ice-cold Dounce homogenizer. After centrifugation at 2,500xg for 10min, the supernatant was collected, and loaded onto a linear 12–48% (w/v) OptiPrep iodixanol gradient prepared according to the manufacturer’s instructions (ProteoGenix). Intracellular compartments and organelles were separated from whole cell lysate by ultracentrifugation at 100,000xg for 18h at 4°C. Twenty-three consecutive fractions of a 100µL volume were collected from the top of the gradient, and then analyzed by SDS-PAGE followed by Western blotting.

### Extracellular vesicles (EVs) purification

BSR, PT or KC cells seeded in 10 cm tissue culture dishes were infected with BTV-1 at an MOI of 1, 10 or 20 respectively for 24h in the EV-depleted conditioned growth medium prepared by ultracentrifugation at 100,000xg for 20h. Cell culture supernatants were collected, centrifuged at 300xg and 3,000xg for 15min each to pellet the detached cells (P1) and dead cells (P2). The supernatant was then centrifuged at 10,000xg for 2h to pellet the LEVs (P3), and then incubated with PEG-10,000 to a final concentration of 8% PEG-10,000 (v/v) on ice for overnight. A final centrifugation at 3000xg for 15min, pelleted the SEVs (P4). The viruses which remained in the supernatant were treated as free supernatant viruses. Each pellet was washed and resuspended in 50µL of PBS.

### Western immunoblotting

Denatured EV protein samples were separated by 10% SDS-PAGE and transferred to PVDF membranes. After blocking in 5% skimmed milk (Millipore) dissolved in TBS (50mM Tris-HCl, pH8.3, 150mM NaCl) with 0.1% Tween (TBST), membranes were incubated with anti-VP2 and anti-NS3 (in-house antibodies), anti-TSG101 (Abcam, ab30871), anti-HSP90 (Proteintech, 11405–1-AP), anti-LAMP1 (Abcam, ab24170), anti-LC3 (Novus, NB600–1384), anti-EDEM1 (Proteintech, 26226–1-AP), and anti-SMPD2 (Proteintech, 15239–1-AP) antibodies diluted 1:1000 in blocking buffer overnight at 4°C, followed by incubation with the appropriate alkaline phosphate (AP)-conjugated secondary antibodies diluted 1:5000 in blocking buffer. 1-Step NBT/BCIP (Thermo Scentific) was then added to the membrane to visualize the target proteins.

### Co-immunoprecipitation (Co-IP)

BSR cells were infected with wild-type or N150A and KKE_**196–198**_/AAA NS3 mutant viruses at an MOI of 1 for 24h prior to lysing in a non-denaturing cell lysis buffer (100mM Tris-HCl, pH 8, 150mM NaCl, 1mM EDTA, 1% NP-40). Mouse anti-NS3 antibody (in-house antibody) was added to cell lysate to allow binding to NS3. Protein G agarose beads (Merck) were then added to allow antibody binding prior to the elution of the bound proteins by subjecting the beads to boiling. VP2, NS3 or GAPDH was detected by western blot according to the previously stated method ‘western immunoblotting’.

### Viral genome quantification

Viral RNA was extracted from isolated LEVs, SEVs and free supernatant virus using the QIAamp Viral RNA kit (Qiagen) following the manufacturer’s instruction. A total of 10µL of eluate was used for cDNA synthesis using the RevertAid reverse transcriptase (Thermo Fisher) with a BTV S6 (NS1) – specific primer (5’-GTAAGTTGAAAAGTTCTAGTAG-3’). A total of 1µL of 1:5 diluted cDNA was then used for real-time quantitative PCR (qPCR) using the PowerUp SYBR Green master mix (Applied Biosystems) with primers (forward: 5’-GGACGATACCGGATTGGAATAA-3’, reverse: 5’-CATCGTAGCATAAGCCCTCTC-3’) targeting the S6, following the manufacturer’s instruction. The viral genome copy number was determined by qRT-PCR.

### Ultrathin sectioning transmission electron microscopy (TEM)

BSR and PT cells seeded on glass coverslips before being infected with BTV-1 at an MOI of 1 and 10 for 24h. Monolayers were fixed in a solution of 2% formaldehyde, 1.5% glutaraldehyde and 100mM sodium cacodylate (pH 7.4) for 1h at room temperature. Cells were dehydrated in increasing concentrations of ethanol (70% ethanol 15min, 90% ethanol 15min, 100% ethanol 15min, 100% ethanol 15min, 100% ethanol 30min, 100% ethanol 45min), and embedded in epoxy resin. Ultrathin slices were cut, stained with Reynolds lead citrate and mount onto carbo-coated nickel grids. Images were collected with a JEM1400 transmission electron microscope (JEOL UK).

### Immunogold labelling

Infected cells were fixed with 4% paraformaldehyde followed by 2% formaldehyde, 0.1% glutaraldehyde and 0.2% HEPES for 1h, and post-fixed in 1% osmium tetroxide, 0.1% sodium cacodylate for 2h. Cells were dehydrated in increasing concentrations of ethanol (70% ethanol 15min, 90% ethanol 15min, 100% ethanol 15min, 100% ethanol 15min, 100% ethanol 30min, 100% ethanol 45min), and embedded in epoxy resin. Ultrathin slices were cut and mounted onto carbon-coated nickel grids. The samples were incubated for 30min in blocking buffer (PBS, 0.1%BSA), followed by overnight incubation at 4°C or 1h at room temperature with mouse anti-NS3 antibody (1:250). After three washes with PBS, 5nm-gold-conjugated anti-mouse secondary antibody (1:50) were incubated for 1h at room temperature and washed three times with PBS.

### Inhibitor assays

BSR cells were infected with BTV-1 at an MOI of 1. At 1h pi, cell culture medium was replaced with the medium containing 10µM of exosome inhibitor GW4869 (Sigma) or 10µM of ERAD inhibitor Eeyarestatin I (EerI) (Cayman Chemical). At 24h pi, cell culture supernatants were harvested for isolation of EVs according to the previous stated method ‘extracellular vesicles purification’.

## Supporting information

S1 Fig Confocal imaging shows BTV NS3 colocalized with outer capsid VP2 and VP5 proteins in close association with the viral VIBs formed by the multimerization of NS2 protein in the early hours (6-8h) pi. NS3, VP2, VP5 and NS2 were probed using specific antibodies with different colours for the different groups. The insets at the bottom right corner show an enlarged version of individual VIBs staining from the area enclosed by the dashed line where the colocalization appears yellow on merged images. Scale bar = 5µm.
(TIF)

S1 Dataset Excel files contains all raw data required to replicate the results of Figures 2B, 3B, 4B, 4D, 5E, 6C, 9B and 9C. (XLSX)
