# Supplementary figures and images for: Insect-borne non-enveloped bluetongue virus utilizes discrete small vesicles for non-lytic release and cell-to-cell transmission
Source: PLoS Pathog. 2025 Oct 9;21(10):e1013582. doi: 10.1371/journal.ppat.1013582 (PMC12578325; doi:10.1371/journal.ppat.1013582)

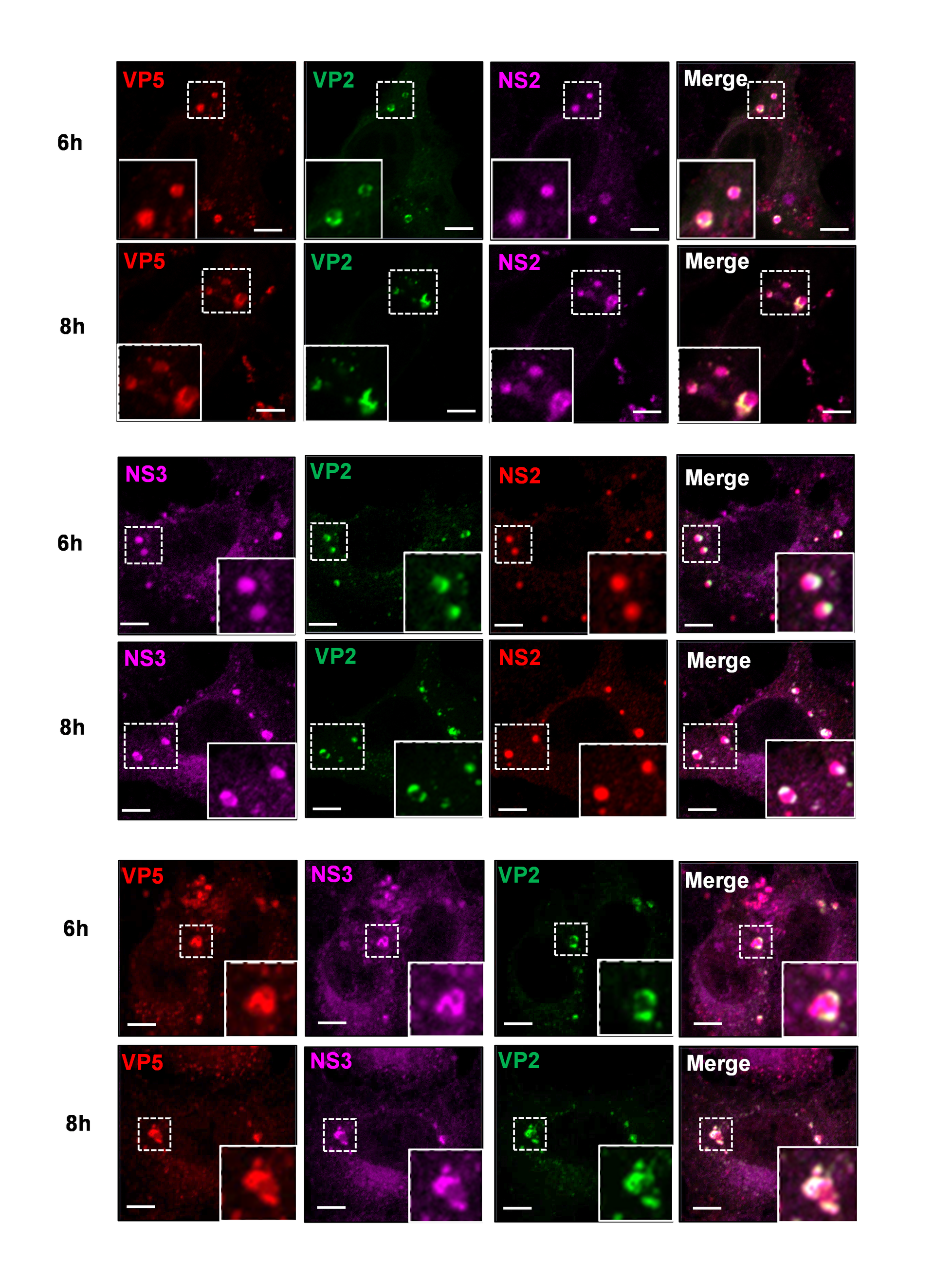

Supplement: S1 Fig — NS3, VP2, VP5 and NS2 were probed using specific antibodies with different colours for the different groups. The insets at the bottom right corner show an enlarged version of individual VIBs staining from the area enclosed by the dashed line where the colocalization appears yellow on merged images. Scale bar = 5µm. (TIF) [file ppat.1013582.s001.tif]
